# Genetic heterogeneity in epilepsy and comorbidities: insights from Pakistani families

**DOI:** 10.1186/s12883-024-03671-7

**Published:** 2024-05-23

**Authors:** Muhammad Yasin, Laura Licchetta, Niamat Khan, Irfan Ullah, Zakir Jan, Muhammad Dawood, Asif Naveed Ahmed, Arfa Azeem, Raffaella Minardi, Valerio Carelli, Shamim Saleha

**Affiliations:** 1 Department of Biotechnology and Genetic Engineering, Kohat University of Science and Technology, Kohat, Khyber Pakhtunkhwa 26000 Pakistan; 2 RCCS, Istituto delle Scienze Neurologiche di Bologna, Bologna, Italy; 3 Department of Neurology, Khyber Teaching Hospital, Peshawar, Khyber Pakhtunkhwa 25000 Pakistan; 4 Department of Neurology, Pakistan Institute of Medical Science, Islamabad, 44000 Pakistan; 5 Department of Biomedical and Neuromotor Sciences, University of Bologna, Bologna, Italy

**Keywords:** ASM-resistant epilepsy, Comorbidities, Genetic heterogeneity, Clinical profiles, Pakistani families

## Abstract

**Background:**

Epilepsy, a challenging neurological condition, is often present with comorbidities that significantly impact diagnosis and management. In the Pakistani population, where financial limitations and geographical challenges hinder access to advanced diagnostic methods, understanding the genetic underpinnings of epilepsy and its associated conditions becomes crucial.

**Methods:**

This study investigated four distinct Pakistani families, each presenting with epilepsy and a spectrum of comorbidities, using a combination of whole exome sequencing (WES) and Sanger sequencing. The epileptic patients were prescribed multiple antiseizure medications (ASMs), yet their seizures persist, indicating the challenging nature of ASM-resistant epilepsy.

**Results:**

Identified genetic variants contributed to a diverse range of clinical phenotypes. In the family 1, which presented with epilepsy, developmental delay (DD), sleep disturbance, and aggressive behavior, a homozygous splice site variant, c.1339–6 C > T, in the *COL18A1* gene was detected. The family 2 exhibited epilepsy, intellectual disability (ID), DD, and anxiety phenotypes, a homozygous missense variant, c.344T > A (p. Val115Glu), in the *UFSP2* gene was identified. In family 3, which displayed epilepsy, ataxia, ID, DD, and speech impediment, a novel homozygous frameshift variant, c.1926_1941del (p. Tyr643MetfsX2), in the *ZFYVE26* gene was found. Lastly, family 4 was presented with epilepsy, ID, DD, deafness, drooling, speech impediment, hypotonia, and a weak cry. A homozygous missense variant, c.1208 C > A (p. Ala403Glu), in the *ATP13A2* gene was identified.

**Conclusion:**

This study highlights the genetic heterogeneity in ASM-resistant epilepsy and comorbidities among Pakistani families, emphasizing the importance of genotype-phenotype correlation and the necessity for expanded genetic testing in complex clinical cases.

**Supplementary Information:**

The online version contains supplementary material available at 10.1186/s12883-024-03671-7.

## Background

Epilepsy is a neurological disease characterized by the presence of recurrent and unprovoked seizures, reflecting underlying brain dysfunction. While seizures are the hallmark of epilepsy, the clinical landscape is often further complicated by the presence of comorbidities—additional medical, cognitive, or behavioral conditions that frequently accompany epilepsy [1, 2]. These comorbidities introduce complexity to the clinical landscape, making it challenging to understand and manage the overall situation. They often lead to more severe phenotypes in epilepsy patients and are associated with negative outcomes, including a poor prognosis, inadequate responses to ASMs, reduced quality of life, and increased mortality [3]. The intricate relationship between epilepsy and its associated comorbidities presents a formidable challenge in neurological research, especially in populations with limited access to advanced diagnostic techniques. In such settings, understanding the genetic components of epilepsy and its associated comorbidities is crucial for effective diagnosis and management. This challenge is particularly relevant in the context of the Pakistani population, characterized by financial limitations and geographical obstacles. Despite the lack of readily available genetic testing services in Pakistan, and the substantial costs associated with high-throughput approaches such as WES, investigating the genetic architecture of epilepsy and associated comorbidities remains imperative. It is essential for a comprehensive understanding of disease pathophysiology and the development of possible precision medicine-based approaches for better clinical management.

Importantly, the occurrence of comorbidities in epileptic patients reflects an overlap of causative genes and the involvement of similar molecular pathways [4, 5]. Previous research has provided convincing evidence that specific genetic variations may play a role in both epilepsy and its associated conditions within individuals. For instance, variants in genes such as *NRXN1, SCN1A, PTEN, TBCD, ARFGEF1, GNAI1, SYNGAP1, GRIN2A, SPTBN5*, and *CNTNAP2* have been linked to epilepsy as well as neurobehavioral and neurodevelopmental comorbidities or other related symptoms [1, 6–15]. The range of comorbidities linked to epilepsy is diverse; a single gene variant may lead to outcomes such as developmental delay (DD), intellectual disability (ID), and various other manifestations, each with differing severity. Additionally, it has been noted that within the same family, individuals carrying identical genetic variants may exhibit a spectrum of diverse phenotypes. For instance, one family member may experience severe, treatment-resistant seizures indicative of ASM-resistant epilepsy, while another may present with milder forms of epileptic seizures [6]. This highlights the complex nature of ASM-resistant epilepsy and its associated conditions, which can be influenced by genetic factors, impacting various aspects of an individual’s health and development.

Therefore, studies investigating familial occurrences of epilepsy may reveal genotypic and phenotypic heterogeneity of epilepsy as well as its comorbidities. Few studies have found an association of genetic variants with epilepsy and its comorbid conditions in families of Pakistani origin. In one study, a missense *CNTNAP2* variant was identified in two consanguineous Pakistani families with epilepsy, DD, ID, and aggressive behavior. Similarly, in another study, a missense *SPTBN5* variant was detected in a Pakistani family with early-onset epilepsy, behavioral impairments, and gastroesophageal reflux [7]. Recently, homozygous variants in *OCLN*, *ALDH7A1*, *IQSEC2*, *COL3A1*, *CNTNAP2*, *TRIT1*, and *NARS1* were identified in individuals from Pakistani families who presented with epilepsy and frequently accompanied DD phenotype [8]. These studies have unveiled a notable lack of genetic investigations specifically targeting families of Pakistani origin from the Khyber Pakhtunkhwa region. This highlights the urgent need to prioritize this geographical area in future research efforts to comprehensively understand the genetic aspects of epilepsy and its management within this population subset. Therefore, in current study, we focused primarily on investigating the genetic causes of epilepsy and its associated comorbidities within four Pakistani families from the Khyber Pakhtunkhwa region. Our investigation revealed diverse clinical phenotypes and distinct homozygous genetic variants associated with specific sets of symptoms, emphasizing the challenging nature of ASM-resistant epilepsy among family members residing in this region.

## Methods

### Study population

The current study was approved by the Ethical Committee as well as the Advance Studies and Research Board of Kohat University of Science and Technology. Families residing in Khyber Pakhtunkhwa, Pakistan, with at least two affected members were eligible for inclusion in the study. The diagnosis of epilepsy was made by Pakistan Medical and Dental Council (PMDC) recognized and qualified epileptologists/neurologists at Khyber Teaching Hospital in Peshawar. It relied primarily on clinical features, including history and examination, with electroencephalography (EEG) as the diagnostic test. Patients resistant to successive lines of drug treatment were classified as ASM-resistant in accordance with the criteria established by the International League Against Epilepsy (ILAE) [9], with consideration given to related variations where indicated. After obtaining written informed consent for genetic analysis, blood samples were collected from all the affected and unaffected family members included.

### Genetic studies

Whole Exome Sequencing (WES) was performed on Genomic DNA of affected individuals (III:4 and III:6 in family1, IV:6 in family2, III:1 in family3 and III:2 in family4). Libraries for each individual were obtained using the xGen DNA Library Prep Kit EZ. Subsequently, they were enriched using the xGen™ Exome Hyb Panel v2 and xGen™ Hybridization and Wash Kit, following the manufacturer’s instructions. Enriched libraries were finally sequenced on the Illumina Novaseq platform, using 151 bp paired-end reads. Sequence data in the form of BAM files were generated via the Picard data-processing pipeline and contained well-calibrated reads aligned to the GRCh37 human Genome reference. Additionally, quality control (QC) statistics for the WES run were assessed (Supplementary Data File 1: Table). Samples across projects were then jointly called via the Genome Analysis Toolkit (GATK) best-practice pipeline31 for data harmonization and variant discovery. This pipeline detected single-nucleotide variants (SNVs) and small insertion or deletion (indel) variants from exome sequence data [10]. All the variants were screened according to the location, frequency, and type of variation. Variants were filtered with a minor allele frequency (MAF) cutoff of < 0.01% in the Exome Variant Server (http://evs.gs.washington.edu/EVS/), GnomAD (https://gnmad.broadinstitute.org), and 1000 Genomes (http://www.1000genmes.org/).

Alleles specific primers (Supplementary Data File 2: Table) were designed using primer3 software (https://www.bioinformatics.nl/cgi-bin/primer3plus/primer3plus.cgi) for the validation and segregation analysis, which involved polymerase chain reaction (PCR) and Sanger sequencing. The amplified PCR products were sequenced by Beijing Tsingke Biotech Co., Ltd. (https://tsingke.com/pages/about-us-1). The Sanger sequencing analysis was carried out using Chromas Lite version 2.6.6, a chromatogram viewer software. The pathogenicity of segregating genetic variants was predicted using various in silico tools: PredictSnp (https://loschmidt.chemi.muni.cz/predictsnp/), Sift (https://sift.bii.a-star.edu.sg/), Mutation assessor (http://mutationassessor.org/r3/) Mutation taster (https://www.mutationtaster.org/), SpliceAI (https://spliceailookup.broadinstitute.org/) and MaxEntScan (http://hollywood.mit.edu/burgelab/maxent/Xmaxentscan_scoreseq.html) SPiP (https://sourceforge.net/projects/splicing-prediction-pipeline/). PhyloP scores for measuring evolutionary conservation were obtained from the UCSC Genome Browser (https://genome.ucsc.edu/). Genetic variants were classified and interpreted using the American College of Medical Genetics and Genomics (ACMG) standards and guidelines [11]. In order to provide a clearer insight into the impact of the variants on the patients’ clinical condition, all reported genetic variants were retrieved from ClinVar (https://www.ncbi.nlm.nih.gov/clinvar/) HGMD (http://www.hgmd.cf.ac.uk/ac/search.php), PubMed (https://www.ncbi.nlm.nih.gov/pubmed/), and OMIM (https://www.ncbi.nlm.nih.gov/omim/) databases.

## Results

In current study, we observed a significant genetic heterogeneity in four Pakistani families with ASM- resistant epilepsy and associated comorbidities, revealing noteworthy clinical phenotype variations both within and between families. The comorbid conditions identified in affected individuals from four epilepsy families are displayed in Table 1. It was determined that these comorbidities had an underlying genetic etiology and were associated with autosomal recessive inheritance of *COL18A1*, *ZFYVE26*, *UFSP2*, and *ATP13A2* variants. Table 2 displays the localization, frequency (gnomAD), predictions, PhyloP scores, pathogenicity (ACMG class), and novelty status of segregating genetic variants. These variants were found within long-sized regions of homozygosity (Supplementary Data File 3: Tables) and demonstrated associations with distinct sets of symptoms and clinical phenotypes.

**Table 1:** Characteristics of individuals in Pakistani families affected with epilepsy and its comorbidities

Investigated Families	Location	Patient ID	Age in years	Age of onset	Seizure type	Comorbidities	Segregating Genetic Variant
Family-1	Orakzai	III:2	25	2 y	GMS	DD, insomnia, anxiety and aggressive behavior	*COL18A1*: c.1339–6 C > T
		III:3	13	2 y	GMS	DD	
		III:4	20	5 y	GMS	DD, insomnia, anxiety, and aggressive behavior	
		III:6	24	3 y	GTCS	DD, insomnia, anxiety and aggressive behavior	
		III:7	10	3 y	GMS	DD	
		III:8	14	2 y	GMS	DD	
		III:9	16	2 y	GMS	DD	
Family-2	Kohat	IV:2	25	4 m	GMS	DD, ID, and anxiety	*UFSP2*: c.344T > A
		IV:5	21	14 y	GTCS	DD, ID, and anxiety	
Family − 3	Kohat	I:1	60	16 y	GTCS	Ataxia	ZFYVE26: c.1926_1941del
		III:1	17	1 m	GTCS	Ataxia, ID, DD	
Family-4	Hangu	III:1	4	1 m	GMS	Speech impediment and deafness, Parkinson’s disease	ATP13A2: c.1208 C > A
		III:2	8	1 m	GMS	ID, DD, Parkinson’s disease, deafness, drooling, speech impediment, hypotonia, and a weak cry	

Y: years, m: months, GMS: Generalized myoclonic seizure, GTCS: Generalized clonic tonic seizure, DD: Developmental delay, ID: Intellectual disability

**Table 2:** Genetic variants with corresponding variant localization, frequency (gnomAD), *in silico* predictions, PhyloP scores, pathogenicity classifications (ACMG class), and novelty status

Genetic Variant	Variant Localization	Frequency (gnomAD)	In silico prediction	PhyloP score	Pathogenicity (ACMG class)	Novelty Status
NM_030582.3 (*COL18A1*): c.1339–6 C > T, p.? (ENSG00000182871)	Splice acceptor site of the intron 4	Overall: **0.0000323** South Asian: **0.0000654** Homozygotes:**0**	Likely Benign	-0.902	Uncertain Significance (PP1, PM2)	Novel
NM_018359.3(*UFSP2*): c.344T > A, p. Val115Glu (ENSG00000109775)	Exon 5	Overall: **0.0000713** South Asian: **0.000847** Homozygotes:**0**	Likely pathogenic	6.331	a. Pathogenic b. (PS3, PM1, PP1, PP2, PP4, PP5)	PMID: 33,473,208
NM_015346.4 (ZFYVE26): c.1926_1941del, p. Tyr643MetfsX2 (ENSG00000072121)	Exon 11	Overall: **0** South Asian:**0** Homozygotes:**0**	Likely pathogenic	2.421	Likely Pathogenic (PVS1)	Novel
NM_022089.4(ATP13A2): c.1208 C > A, p. Ala403Glu (ENSG00000159363)	Exon 13	Overall: **0.0000297** South Asian: **0.0005271** Homozygotes: **0**	Damaging	4.442	a. Uncertain Significance (PM1, PM2, PP1)	Reported in ClinVar (Accession #: SCV000351396)

**Fig. 1:**
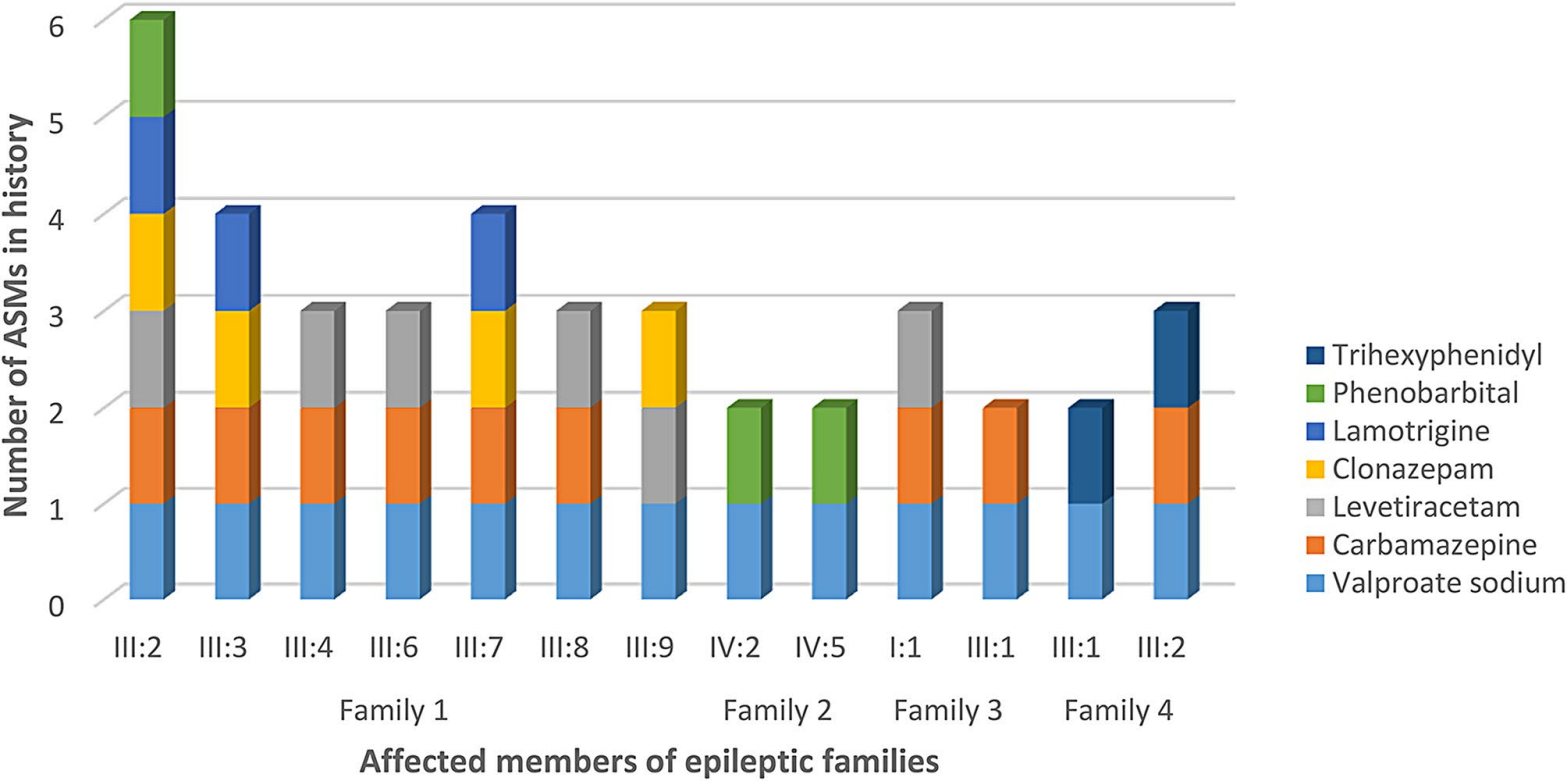
The number of ASMs used in history by the epileptic patients in investigated families

**Fig. 2:**
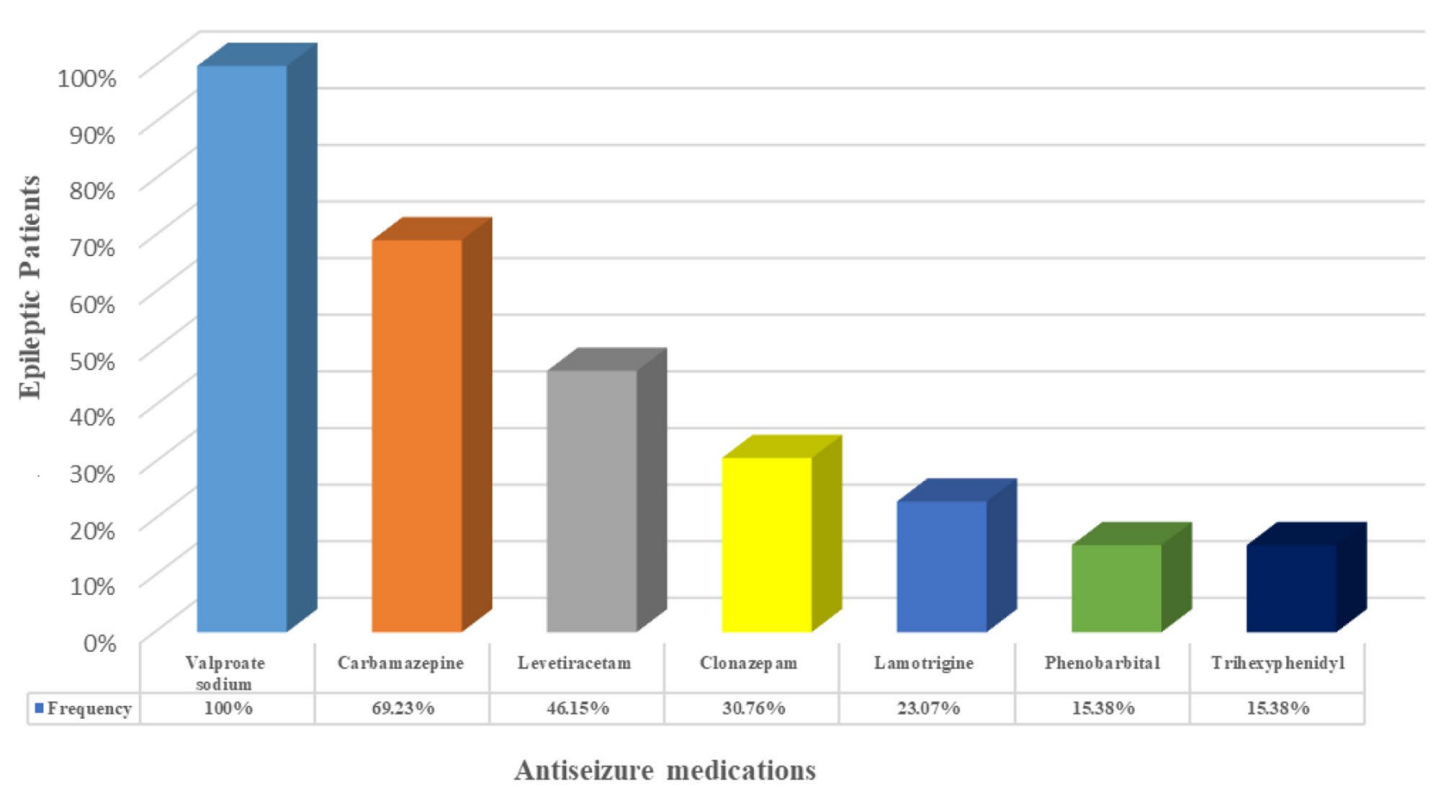
The frequency of ASMs usage by epileptic patients in investigated families

Figure 1 displays the number of ASMs used in history by the epileptic patients in the investigated families. It’s noteworthy that these patients were prescribed multiple ASMs in their treatment efforts, yet their seizures persisted, indicating cases of ASM-resistant epilepsy. Similarly, Fig. 2 depicts the frequency of ASMs usage by epileptic patients in investigated families, and notably, Valproate Sodium was the most used ASM by Pakistani epileptic patients.

### Family 1

The proband of family-1 was a 20-year-old male (III:4) with epilepsy, DD, insomnia, and aggressive behavior born from non-consanguineous parents. He experienced the first unprovoked seizure at the age of 5 years. His seizures control could not be achieved with prescribed ASMs. The interictal EEG shows generalized myoclonic seizures. The affected siblings (III:2, III:3 & III:4) and cousins (III:6, III:7, III:8 & III:9) were exhibiting similar disease features. DD during childhood was a prevalent feature among them. Furthermore, as individuals aged, they exhibited symptoms like insomnia and increased aggression (Table 1).

WES analysis revealed a novel homozygous splice site variant [Chr21 (GRCh37): g.46,896,259 C > T NM_001379500.1: c.1339–6 C > T] in *COL18A1* in the proband. *In silico* analysis predicted it as ‘likely benign,’ as the PhyloP score indicated a low level of evolutionary conservation at this position. However, Sanger sequencing verified the segregation of the *COL18A1* variant with the observed phenotype, confirming its homozygous status in the proband, his siblings, and cousins (Fig. 3.1a & b). This variant consistently manifested as a recessive allele in affected family members and was never observed in a homozygous state within the control population database. The rarity of this variant in its monoallelic status in GnomAD (MAF = 0.0000323) emphasizes its potential as a cause of an autosomal recessive disorder (Table 2). Based on these factors, the COL18A1 c.1339–6 C > T variant is classified as a Variant of Uncertain Significance (VUS) in accordance with ACMG guidelines (Table 2).

**Fig. 3:**
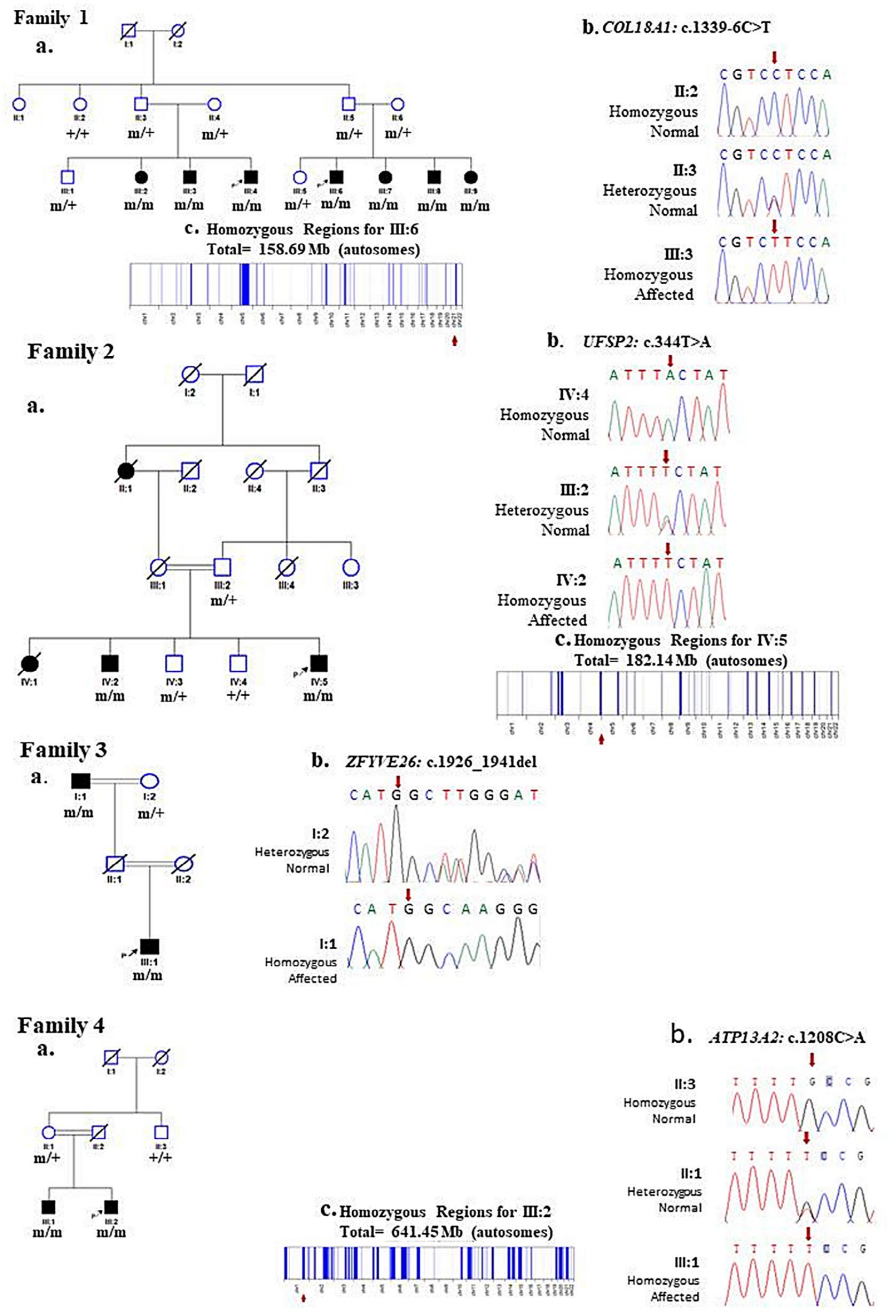
Families pedigrees and genetic findings: **1a**. Pedigree of family 1, **1b**. showing segregation of the identified variant in *COL18A1* gene, **1c.** Homozygosity map of the proband (III:6), showing the runs of homozygosity as blue bands. **2a**. Pedigree of family 2, **2b.** showing segregation of the identified variant in *UFSP2* gene, **2c.** Homozygosity map of the proband (IV:5), showing the runs of homozygosity as blue bands. **3a**. Pedigree of family 3, **3b.** showing segregation of the identified variant in *ZFYVE26* gene. **4a**. Pedigree of family 4, **4b.** showing segregation of the identified variant in *ATP13A2* gene, **4c.** Homozygosity map of the proband (III:2), showing the runs of homozygosity as blue bands

### Family 2

The proband (IV:5) in family-2, a 21-year-old male was born from consanguineous healthy parents (Fig. 3.2a). The proband experienced the first unprovoked seizure at the age of 14 years. In addition, he did not respond to ASM. Interictal EEG shows generalized clonic tonic seizure. Brain computed tomography scan was normal (Table 1). Epilepsy-associated comorbidities observed in the proband included DD, ID, and anxiety. His 25-year-old brother has similar findings (Table 1). His sister (IV:1) and aunt (II:1) both deceased due to status epilepticus during childhood.

By WES analysis, a homozygous missense variant [Chr4(GRCh37): g.186,337,011 A > T, NM_018359.5: c.344T > A, p. Val115Glu] in *UFSP2* gene was identified in the proband. The segregation study verified the inheritance pattern of the *UFSP2* variant, confirming its homozygous status in both the proband and his brother (Fig. 3.2a & b). The variant c.344T > A in *UFSP2* gene with frequency (MAF = 0.0000713) was listed in the gnomAD that leads to a substitution of Valine with Glutamic Acid and at the evolutionary conserved position 115 (p. Val115Glu) according to the UCSC Human Genome (GRCh37/ hg19) and Ensemble database. In addition, this variant is predicted as pathogenic by ACMG and likely pathogenic by in silico analysis (Table 2). The variant has already been described in the literature as pathogenic and associated with pediatric neurodevelopmental anomalies and epilepsy phenotype (PMID: 33,473,208).

### Family 3

In Family-3, a proband, a 7-year-old child (III:1), who presented a complex clinical phenotype. The proband’s medical history, as revealed through the interictal EEG examinations, indicated the onset of generalized tonic-clonic seizures at an exceptionally early age of one month. Additionally, the proband exhibited symptoms of epilepsy, ataxia, ID, and DD (Table 1). Notably, both parents were phenotypically normal, despite their consanguineous relationship. The grandfather (I:1) of the proband had similar features of epilepsy and ataxia but did not exhibit ID and DD (Fig. 3.3a).

WES analysis was conducted, revealing a novel homozygous frameshift variant, [Chr14 (GRCh37): g.68265038GGCTTGGCATTGTATAA > G NM_015346.4: c.1926_1941del], in the *ZFYVE26* gene within the proband. The validation studies verified its homozygous status in both the proband and his grandfather (Fig. 3.3a & b). Based on ACMG criteria and *in silico* analysis, the variant, denoted as c.1926_1941del, is predicted to be a “variant of likely pathogenic”. Remarkably, this variant consistently presented as a recessive allele in all affected family members and was not detected in a homozygous state within control population databases (Table 2). Its absence in its monoallelic status in both the Genome Aggregation Database (GnomAD) and the 1000 Genomes Project further underscores its potential pathogenic role in the context of an autosomal recessive disorder. These findings suggest a compelling link between the *ZFYVE26* variant and the observed clinical phenotypes in the proband and his grandfather, shedding light on its likely pathogenic impact.

### Family 4

Within the scope of this study, we investigated family 4, which presented a remarkable clinical complexity. The proband (III:2), a nearly 10-year-old child, displayed a constellation of symptoms, including epilepsy, Parkinson’s disease (PD), ID, DD, deafness, excessive drooling, speech impediment, hypotonia, and a notably weak cry (Table 1). Seizures first manifested at a remarkably early age of just one month, with interictal EEG reports indicating generalized myoclonal seizures. Intriguingly, the parents of the proband had a consanguineous relationship (Fig. 3.4a), yet both showed no overt phenotypic abnormalities. Within the same family, an affected sibling (III:1) displayed similar clinical characteristics, including epilepsy, Parkinson’s disease, speech impediment, and deafness, but did not experience ID or DD (Table 1).

WES analysis was systematically performed, leading to the identification of a homozygous missense variant, [Chr1 (GRCh37): g.17,322,979 C > A NM_022089.4: c.1208 C > A_p.Ala403Glu], localized within the *ATP13A2* gene, in the proband of the studied family. To validate the inheritance pattern of this *ATP13A2* variant and its connection with the manifested clinical phenotypes, Sanger sequencing was conducted, conclusively affirming its homozygous status in both the proband and a sibling (Fig. 3.4a & b). Based on ACMG criteria and *in silico* analysis, the variant c.1208 C > A, is classified as a VUS and ‘damaging’ respectively (Table 2). Remarkably, this particular genetic alteration consistently demonstrated a recessive mode of inheritance across all afflicted family members and remained conspicuously absent in the homozygous state within population databases utilized for control comparisons. Its rare occurrence in the heterozygous state, as evident in both the GnomAD (MAF = 0.0000297) databases, further underscores its potential implication as a pathogenic contributor within the framework of an autosomal recessive disorder. These findings collectively offer compelling evidence of an association between the *ATP13A2* variant, and the clinical features observed in the proband, thus highlighting its plausible pathogenic role.

## Discussion

Investigation of epilepsy and comorbidities in the Pakistani population has the potential to contribute not only to the advancement of personalized medical approaches but also to the enrichment of our knowledge about the underlying genetic factors and clinical profiles associated with this challenging neurological condition. Typically, family history, clinical assessments, and investigations using EEG/video-EEG telemetry and brain imaging (such as computed tomography or magnetic resonance imaging) can establish a clinical diagnosis in epilepsy patients. Nevertheless, many of these clinical diagnostic approaches are beyond the reach of patients in remote regions of Pakistan due to financial limitations and a lack of availability at their residential areas. Moreover, the presence and variable phenotypes of comorbidities in patients with epilepsy pose significant challenges for diagnosis and management [12]. Importantly, the use of next-generation sequencing (NGS) technologies, such as WES, is extremely useful in determining the underlying genetic causes of epilepsy and its comorbid conditions in families. Using WES, homozygous pathogenic variants in the *COL18A1, UFSP2, ZFYVE26* and *ATP13A2* genes were detected to cause ASM-resistant epilepsy and its comorbid conditions in four Pakistani families. While acknowledging the limitations and costs associated with genetic testing, particularly in resource-limited settings, our findings emphasize the importance of genetic investigations in elucidating the molecular basis of ASM-resistant epilepsy and guiding precision medicine-based approaches for improved clinical management.

The *COL18A1* gene encodes the α1 chain of collagen type XVIII, which is a component of basement membranes, specialized structures critical for the normal development of various tissues [13]. Different tissues have specific requirements for collagen composition and organization [14]. Importantly, Collagen XVIII is necessary for the normal development of the brain and eye. Consequently, disrupted function of this protein can result in central nervous system (CNS) and eye defects, respectively [13]. Biallelic variants in the *COL18A1* gene were first described to cause a very rare autosomal recessive syndrome known as Knobloch syndrome (KS), characterized by a range of ocular, genitourinary and CNS defects [14]. Since the first report, many studies have associated *COL18A1* variants with KS (Supplementary Data File 4: Figure) and have observed variations in phenotype among patients with KS [15–17]. These studies reported KS patients from different ethnicities who most commonly displayed ocular defects such as myopia, cataract, lens subluxation, retinal detachment, vitreoretinal degeneration, retinitis pigmentosa, and glaucoma [13, 18]. They rarely exhibited occipital defects such as encephalocele and CNS defects like cognitive decline, autism, epilepsy, developmental delay, delayed wound healing, and renal abnormalities [17, 19–21]. Previous studies detected pathogenic *COL18A1* variants in patients with confirmed clinical diagnoses of KS [22]. Furthermore, studies have reported patients carrying pathogenic *COL18A1* variants for whom a syndromic diagnosis was not initially made, but who exhibited hallmark CNS defects with or without ocular and occipital defects seen in KS [23, 24]. However, García-Prieto and colleagues reported a patient carrying pathogenic *COL18A1* variants with brain defects such as autism and DD, without other significant ocular and occipital defects [21]. Similarly, the individuals under investigation in the present study were diagnosed with epilepsy along with its comorbid conditions, including developmental delay, sleep disruptions, and aggressive behavior. Moreover, it was observed that sleep disturbances and aggressive behavior were associated with age, while developmental delay seemed to be a characteristic of childhood. While a clinical diagnosis of KS was not applicable due to the absence of typical ocular and occipital defects in affected individuals, our findings, alongside previous research, demonstrate that *COL18A1* variants such as c.193G > A (p.Gly65Arg) and c.3150G > A (p.Trp1050Term) are associated with sudden unexpected death in epilepsy [25]. Moreover, *COL18A1* variants c.3690G > A (p.Trp1230*) and c.4063_4064delCT (p.Leu1355Valfs*72) have been linked to familial epilepsy with anterior polymicrogyria [22]. Additionally, a variant c.3514-3515delCT has been associated with an autosomal recessive disorder characterized by epilepsy, ataxia, cognitive decline, and visual problems [26]. These findings of present and previous studies highlight the intricate interplay between genetic variations in *COL18A1* and the development of epilepsy and associated conditions.

Individuals may experience different combinations of disease features depending on the effects of specific *COL18A1* variants on the protein’s structure and function. Among the reported pathogenic *COL18A1* variants in homozygous and compound heterozygous states in KS families in previous studies, the majority of them were described as loss-of-function variants causing protein truncation, leading to KS [15, 18, 27]. Previously, compound heterozygous *COL18A1* variants were identified in patients with CNS defects such as autism and developmental delay without ocular malformations. Expression analysis in these studies revealed that residual expression of *COL18A1* in eye cells allows normal ocular development. Thus, it was inferred that compound heterozygous *COL18A1* variants do not completely abolish COL18A1 expression [21, 28, 29], but they may impact the expression of COL18A1 in different tissues, potentially developing a correlation between disease phenotype and tissues expressing COL18A1. In the current study, the *COL18A1* variant c.1339–6 C > T was initially classified as a VUS according to the ACMG criteria. This variant was consistently found in a total of seven affected individuals within the family in a homozygous state, which is a significant finding. Conversely, it was absent in unaffected family members in the homozygous state, as well as in the gnomAD database, with an overall MAF of 0.0000323. These factors collectively provide strong evidence for its classification as a VUS. Thus, the current study provides evidence that the *COL18A1* variant might cause ASM-resistant epilepsy and comorbidities. In the current study, a VUS in the *COL18A1*gene was found to be associated with disease phenotypes in a Pakistani family, possibly causing ASM-resistant epilepsy and comorbidities. Further research, including genotype-phenotype correlation and functional/expression studies, is necessary to fully understand the significance of the identified *COL18A1* variant in disease manifestation.

The *UFSP2* gene encodes a cysteine protease that participates in Ubiquitin-fold modifier 1 (UFM1) maturation in a process known as UFMylation [30] and also releases UFM1 from UFMylated proteins in a process known as de-UFMylation [31, 32]. Both UFMylation and de-UFMylation are processes of post-translational protein modification, and functional studies have revealed that the loss of key components of these processes results in defects in embryogenesis, hematopoiesis, cellular differentiation, and brain development [30, 33]. Pathogenic variants (p. Asp426Ala, p.His428Arg, p.Cys302Ser) in two of the three components of the catalytic triad (Asp426 and His428) have already been associated with patients with different types of skeletal dysplasia [34–36]. Homozygous variant (p.Val115Glu) were found to cause drug-refractory epilepsy and other brain defects [33, 37]. Specifically, p.Val115Glu was found to cause drug-refractory epilepsy and DD, whereas the p.Val115Glu variant was detected in patients with drug-refractory epilepsy, infantile spasms, and severe ID [33]. In the current study, the p.Val115Glu variant was found to cause ASM-resistant epilepsy, DD, ID, and anxiety in patients of a Pakistani family. Ni and colleagues’ study findings suggested that variants observed in human skeletal dysplasia impact UFSP2’s catalytic activity, whereas the homozygous p.Val115Glu variant found associated with brain defects impacts the N-terminal domain, including protein stability and interaction with UFMylated targets. Thus, the findings of the current study and previous studies performed by Ni et al. (2021) and Raha et al. (2023) provide significant evidence for the association of homozygous *UFSP2* variants with brain defects [33, 37].

The *ZFYVE26* encodes a protein known as spastizin, which is a large molecule consisting of 2539 amino acid residues. This protein comprises three distinct domains: a zinc finger domain, a leucine zipper domain, and a FYVE (Fab-1, YGL023, Vps27, and EEA1) domain [38]. Spastizin is a crucial component of the adaptor-related protein complex 5 (AP5), which plays a significant role in the process of autophagic lysosomal reformation. The Z*FYVE26* variants result in disruptions to the fusion of autophagosomes and endosomes [39]. Homozygous variants in Z*FYVE26* have been reported to cause autosomal recessive hereditary spastic paraplegia (AR HSP), which is characterized by progressive spasticity and weakness of the lower limbs, often accompanied by cognitive impairment and a thin corpus callosum on magnetic resonance imaging (MRI) scans [40–42]. In some cases, it may also be accompanied by distal amyotrophy, pigmentary maculopathy, and atypical parkinsonism [43, 44]. Here, we expand the phenotypic spectrum associated with Z*FYVE26* variants, by reporting a Pakistani epileptic family with spastic ataxia DD and ID phenotypes. Most of the variants identified in Z*FYVE26* are frameshift or nonsense variants, indicating a loss-of-function mechanism in the pathogenesis of AR HSP [42]. Similarly, the Z*FYVE26* variant p.Tyr643MetfsX2 identified in the current study is a frameshift variant resulting in a truncated protein, disrupting the normal function of the gene and, consequently, causing ASM-resistant epilepsy, spastic ataxia, DD, and ID phenotypes in patients. The findings of both the previous and current studies expand our understanding of the phenotypic spectrum associated with Z*FYVE26* variants and affirm the idea that the Z*FYVE26* variants primarily result in a loss-of-function mechanism in the development of neurodegenerative and neurodevelopmental phenotypes.

The *ATP13A2* gene encodes a lysosome-associated transmembrane enzyme that restores lysosomal function under physiological conditions. This enzyme shows high expression in the human brain [45] and loss-of-function *ATP13A2* variants have been reported to cause rare autosomal recessive juvenile-onset neurodevelopmental and neurodegenerative conditions. These conditions include Kufor-Rakeb syndrome [46], spastic paraplegia [47], neuronal ceroid lipofuscinoses [48], and amyotrophic lateral sclerosis [49]. In patients with these phenotypes, lysosomal function is impaired due to missense or nonsense *ATP13A2* variants. Recent research has revealed that missense *ATP13A2* variants cause misfolding of the ATP13A2 protein. The misfolding of the ATP13A2 protein relocates it from its original lysosomal position to the endoplasmic reticulum (ER) [50–52]. The ER serves a crucial role in eliminating unwanted and potentially harmful proteins [53]. Once the misfolded ATP13A2 protein is in the ER, it triggers the endoplasmic reticulum-associated degradation (ERAD) pathway, resulting in its rapid degradation. This process ultimately leads to a decrease in *ATP13A2* protein levels within lysosomes [54, 55]. In the case of a nonsense *ATP13A2* variant, the transcribed mRNA triggers the nonsense-mediated mRNA decay (NMD) pathway due to the presence of a premature termination codon. Eventually, the nonsense ATP13A2 mRNA is degraded, also leading to a decrease in ATP13A2 protein levels within lysosomes [51, 56]. The ERAD and NMD pathways serve as the molecular basis for the loss-of-function nature of *ATP13A2* variants.

In the current study, we identified a homozygous missense variant in the *ATP13A2* gene, specifically c.1208 C > A, p.Ala403Glu, in a family displaying a range of symptoms, including ASM-resistant epilepsy, ID, DD, PD, deafness, drooling, speech impediments, hypotonia, and a weak cry. This particular *ATP13A2* variant had previously been classified as a VUS within the context of juvenile-onset PD syndrome (Kufor-Rakeb syndrome) in the ClinVar Miner database (ClinVar; SCV000351396). However, our segregation analysis in a Pakistani family has, for the first time, established the pathogenic nature of this *ATP13A2* variant in relation to the observed disease phenotypes. The variant was consistently identified as a recessive allele in affected individuals and was absent in unaffected individuals. Furthermore, it exhibited an exceedingly low frequency in gnomAD (MAF = 0.0000297), providing compelling evidence of its pathogenicity. Consequently, our study strongly supports the hypothesis that the *ATP13A2* variant is causative for ASM-resistant epilepsy and the associated comorbidities, including PD.

In conclusion, this study highlights the genetic and clinical heterogeneity associated with ASM-resistant epilepsy and its comorbidities in the Pakistani population. Pathogenic variants in *COL18A1*, *UFSP2*, *ZFYVE26*, and *ATP13A2* contribute to a wide range of clinical phenotypes, from ASM-resistant epilepsy and developmental delay to ataxia, intellectual disability, speech impediment, and other associated symptoms. The findings underscore the importance of genotype-phenotype correlation and the need for expanded genetic testing, especially in challenging clinical cases. Addressing financial and geographical barriers to healthcare access in remote regions of Pakistan is essential for improved patient care. Moreover, further research into the functional consequences of specific genetic variants and the establishment of rare disease registries can advance our understanding and support for individuals with epilepsy and comorbid conditions.

### Electronic supplementary material

Below is the link to the electronic supplementary material.

Supplementary Material 1

Supplementary Material 2

Supplementary Material 3

Supplementary Material 4

## Data Availability

The datasets generated and/or analyzed during the current study are available in the ClinVar repository, with the accession numbers i.e., SCV004174822, SCV004174821, SCV004174820, and SCV004174819.
