# Supplementary material for: Genetic heterogeneity in epilepsy and comorbidities: insights from Pakistani families
Source: BMC Neurol. 2024 May 23;24:172. doi: 10.1186/s12883-024-03671-7 (PMC11112905; doi:10.1186/s12883-024-03671-7)
Supplement: Supplementary file 1 — Supplementary Material 1 [file 12883_2024_3671_MOESM1_ESM.docx]

**Supplementary data table: Quality control (QC) statistics for WES run**

| **Chromosome** | **Position** | **Gene Symbol** | **FILTER** | **Total Depth of Coverage** | **Allelic depths** |
| --- | --- | --- | --- | --- | --- |
| chr21 | 46896259 | *COL18A1* | PASS | 222 | 222 |
| chr4 | 1.86E+08 | *UFSP2* | PASS | 65 | 63 |
| chr14 | 68265037 | *ZFYVE26* | PASS | 103 | 103 |
| chr1 | 17322979 | *ATP13A2* | PASS | 183 | 182 |
