# Supplementary material for: Genetic heterogeneity in epilepsy and comorbidities: insights from Pakistani families
Source: BMC Neurol. 2024 May 23;24:172. doi: 10.1186/s12883-024-03671-7 (PMC11112905; doi:10.1186/s12883-024-03671-7)
Supplement: Supplementary file 2 — Supplementary Material 2 [file 12883_2024_3671_MOESM2_ESM.docx]

**Table: Alleles specific primers designed for validation segregation studies**

| **Genetic Variants** | **Primers Pairs (5**′**-3**′ **sequence)** | **Length** | **Tm (^o^C)** | **Product Size (bp)** |
| --- | --- | --- | --- | --- |
| *COL18A1*  c.1339-6C>T, p.? | **F**-GACCCTCTGCAGATTCACATCA | 22 | 60.09 | 385 |
|  | **R**- ACTTCTGGAATCTTCCGTGCT | 21 | 59.37 |  |
| *UFSP2*  c.344T>A,  p. Val115Glu | **F**-GGGAAGGGCCCTCATTCAAA | 20 | 59.96 | 451 |
|  | **R**-TCTGTCCTTTAAAAGTCCTTCTACT | 25 | 57.39 |  |
| *ZFYVE26*  c.1926_1941del | **F**-CTGAGGAAGGCCCCTATTGC | 20 | 60.18 | 306 |
|  | **R**-TCTTCACCCAGAGCCTCACT | 20 | 60.18 |  |
| *ATP13A2*  c.1208C>A,  p. Ala403Glu | **F**: GTCGAGAGCCCGGATTACAA | 20 | 59.54 | 510 |
|  | **R**: GTTAGGACTCCACACTGCTGA | 21 | 59.38 |  |
