# Supplementary material for: Genetic heterogeneity in epilepsy and comorbidities: insights from Pakistani families
Source: BMC Neurol. 2024 May 23;24:172. doi: 10.1186/s12883-024-03671-7 (PMC11112905; doi:10.1186/s12883-024-03671-7)
Supplement: Supplementary file 4 — Supplementary Material 4 [file 12883_2024_3671_MOESM4_ESM.docx]

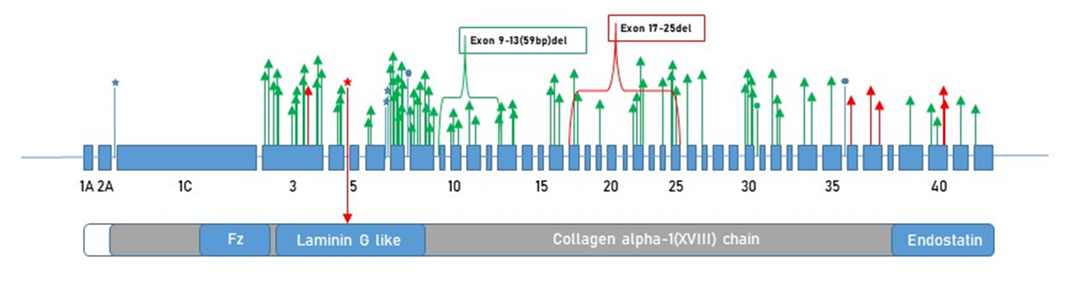


**Figure**: The systematic diagram illustrates the exons and domains of *COL18A1*, with variants highlighted*:*

Splice donor site variants.

A splice acceptor site variant identified in current study.

Splice acceptor site variants.

Epilepsy and associated conditions

Knobloch syndrome
